# SOCIOECONOMIC STATUS AND THE GENDER GAPS IN LATE AGE SELF-REPORTED HEALTH IN TAIWAN

**DOI:** 10.1093/geroni/igz038.1172

**Published:** 2019-11-08

**Authors:** Robert White, Fang-Yi Huang

**Affiliations:** 1 University of Florida, Gainesville, Florida, United States; 2 Trinity Washington University, College Park, Maryland, United States

## Abstract

The socio-economic status (SES) are the fundamental causes of gendered health disparity. However, how the gender gap in self-reported health (SRH) mediate by SES at old age over time is still unresolved. Some argue the SES measures play more and more important role to explain the gender gap in SRH at later age because the feminization of poverty and female’s longer widowhood increased the gap over life course through cumulative disadvantage approach. But others SES-SRH gradients in gender gap keep convergent by age since the effects of SES on health for male has declined and make the gender gap in health disparity vanish over time. Our results show for every age, increasing SES is associated with declining risk of reporting poor health and the effects can explain much more for women than men, especially for the younger old below age 70. The effects of SES and marriage on the magnitude of the gender gap are substantial approximately 40 percent among seventy year olds to the full gender gap among 55-64 year olds in 2006.

